# Demographic, Clinical, Radiological, and Cytopathological Profiles of Patients With Gallbladder Carcinoma in Tripura: A Hospital-Based Cross-Sectional Study

**DOI:** 10.7759/cureus.111129

**Published:** 2026-06-19

**Authors:** Hriday Das, Kaushik Datta, Susmita Dutta, Diptendu Chaudhuri

**Affiliations:** 1 General Surgery, Agartala Government Medical College, Govind Ballabh Pant Hospital, Agartala, IND; 2 Physiology, Tripura Medical College, Agartala, IND

**Keywords:** adenocarcinoma, clinical staging, cytopathology, gallbladder carcinoma, north-east india, tripura

## Abstract

Background

Gallbladder carcinoma (GBC) is an aggressive malignancy characterized by rapid local invasion, delayed clinical presentation, and poor survival outcomes. Despite the high disease burden in North-East India, region-specific data on the demographic and diagnostic characteristics of GBC remain limited.

Aim

This study aims to describe the demographic characteristics, clinical presentation, laboratory abnormalities, radiological findings, cytopathological features, and diagnostic stages among patients with GBC in Tripura.

Materials and methods

This hospital-based cross-sectional study included 192 consecutive patients with cytologically confirmed GBC who were managed at a tertiary-care center in Tripura between October 2024 and September 2025. Demographic characteristics, clinical features, laboratory parameters, imaging findings, cytopathological diagnoses, and clinical stage at presentation were analyzed using descriptive statistics.

Results

A total of 192 patients were included, with a mean age of 61.3 ± 6.1 years. Females constituted 93.8% of the study population, and 56.3% of patients were older than 60 years. Three-fourths of patients belonged to lower-middle, upper-lower, or lower socioeconomic classes and resided in rural areas (75.0%). A non-vegetarian diet was reported by 81.3% of patients, and betel-nut chewing by 71.9%. Abdominal pain and loss of appetite were present in all patients, and significant weight loss was observed in 90.6% of patients. Jaundice and palpable gallbladder were noted in 62.5% and 81.3% of patients, respectively. Anemia was present in 96.9% of patients, and hyperbilirubinemia in 59.4%. Ultrasonography identified a gallbladder mass in 56.3% of patients, and contrast-enhanced computed tomography frequently demonstrated locally advanced disease. Cytopathological evaluation revealed adenocarcinoma in 168 (87.5%) patients. Cytopathological evaluation revealed adenocarcinoma in 168 (87.5%) patients. Clinical staging demonstrated that 75.0% of patients presented with stage III-IV disease, indicating substantial diagnostic delay.

Conclusions

GBC in Tripura predominantly affects elderly women and is commonly diagnosed at an advanced stage. Most patients in this cohort originated from rural and lower socioeconomic backgrounds. The high proportion of advanced-stage disease at presentation highlights the need for improved awareness, earlier diagnostic evaluation of symptomatic patients, and strengthened referral pathways in high-burden regions of North-East India.

## Introduction

Gallbladder carcinoma (GBC) is an aggressive malignancy characterized by high mortality. The absence of a serosal barrier facilitates rapid local invasion and early dissemination of tumor cells, contributing to delayed diagnosis. Consequently, long-term survival remains poor, particularly in advanced-stage disease. The absence of a serosal barrier facilitates rapid local invasion and early dissemination of tumor cells, leading to delayed diagnosis and a five-year survival rate of less than 5% [1]. GBC is the most common malignancy of the biliary tract, accounting for approximately 80-95% of cases, and ranks sixth among gastrointestinal malignancies worldwide [2].

The global incidence of GBC is relatively low, at less than two per 100,000 population; however, marked geographic, gender, and ethnic variations have been documented [3]. In Chilean women, GBC is a leading cause of cancer-related mortality and is associated with particularly poor survival outcomes [4]. In India, the incidence varies widely across regions, with the northeastern states demonstrating one of the highest disease burdens, particularly in the Kamrup district of Assam [5,6].

The population of North-East India differs significantly from that of the rest of the country in terms of ethnicity, dietary habits, literacy levels, lifestyle factors, and geographic characteristics, all of which may influence disease epidemiology. Despite the high burden of disease, published data on GBC from this region remain scarce. Therefore, this study aimed to describe the demographic, clinical, radiological, cytopathological, and staging characteristics of patients with GBC in Tripura.

## Materials and methods

This hospital-based cross-sectional observational study included consecutive patients presenting between 1 October 2024 and 30 September 2025. The study population comprised consecutive patients with cytologically confirmed GBC managed at the Department of General Surgery, Agartala Government Medical College and GBP Hospital (AGMC & GBP Hospital), Agartala, and the Atal Bihari Vajpayee Regional Cancer Centre (ABV-RCC), Agartala, during the study period. Institutional permission was obtained from ABV-RCC to access relevant patient data. Patients identified from both institutions were cross-verified using available demographic and hospital registration details, and duplicate entries were removed prior to analysis to ensure that each patient was included only once.

Eligibility for inclusion required cytological confirmation of GBC by image-guided fine-needle aspiration cytology (FNAC) obtained from the primary gallbladder lesion or another accessible site, including metastatic liver lesions or regional lymph nodes, where appropriate. Patients without cytological confirmation, those who declined participation, and those with a prior history of another malignancy were excluded. Census sampling was employed, and all eligible patients presenting during the study period were enrolled.

Clinical evaluation included laboratory investigations such as liver function tests, hemoglobin level, total leukocyte count, serum urea, and serum creatinine. Serum tumor markers, namely carcinoembryonic antigen (CEA) and carbohydrate antigen 19-9 (CA 19-9), were assessed in all patients. Imaging evaluation included ultrasonography (USG) and contrast-enhanced computed tomography (CECT) of the abdomen.

As definitive surgical resection was not performed in a substantial proportion of patients, pathological staging was unavailable in many cases. Therefore, the clinical stage at presentation was assigned based on available radiological findings in accordance with the 8th edition of the American Joint Committee on Cancer (AJCC) staging system for GBC [7].

Demographic variables including age, sex, education, occupation, socioeconomic status, religion, place of residence, dietary habits, addiction history, previous history of cholecystitis, comorbidities, and family history of cancer were recorded using a predesigned structured pro forma. Clinical, laboratory, radiological, cytopathological, and staging characteristics were documented and analyzed. Socioeconomic status was assessed using the Modified Kuppuswamy Socioeconomic Scale [8].

Data were analyzed using SPSS Statistics version 21.0 (IBM Corp. Released 2012. IBM SPSS Statistics for Windows, Version 21.0. Armonk, NY: IBM Corp.). Continuous variables were summarized using mean ± standard deviation, while categorical variables were expressed as frequencies and percentages. Written informed consent was obtained from all participants prior to enrollment. The study protocol was approved by the Institutional Ethics Committee for Clinical Studies of Agartala Government Medical College (approval number: F.4(6-13)/AGMC/Medical Education/IEC Approval/2022/26148).

## Results

A total of 192 patients with cytologically confirmed GBC were included in the study. The mean age at presentation was 61.3 ± 6.1 years, and more than half of the patients (56.25%) were older than 60 years. A marked female predominance was observed, with females accounting for 93.75% of the study population (female-to-male ratio 15:1).

Most patients had low educational attainment, with 71.9% being either illiterate or educated only to the primary school level. Housewives constituted the largest occupational group (68.8%). Three-fourths of patients belonged to lower-middle, upper-lower, or lower socioeconomic classes according to the Modified Kuppuswamy Socioeconomic Scale. The majority were Hindu (84.4%) and resided in rural areas (75.0%). A non-vegetarian diet was reported by 81.3% of patients. Betel-nut chewing was the most common addiction (71.9%), whereas smoking and alcohol consumption were reported by 6.3% of patients each. A previous history of cholecystitis was documented in 40.6% of cases. Hypertension and diabetes mellitus were present in 25.0% and 12.5% of patients, respectively, while 12.5% reported a family history of cancer in a first-degree relative (Table 1).

**Table 1:** Demographic profile of patients with GBC (n = 192) # Percentages of Variables may not total 100% because of rounding. * Socioeconomic status was determined using the Modified Kuppuswamy Socioeconomic Scale. ** Patients could have more than one addiction; therefore, percentages do not sum to 100%. GBC: gallbladder carcinoma

Variable^#^	Category	Frequency (%)
Age (years)	<40	12 (6.25)
	40-60	72 (37.50)
	>60	108 (56.25)
Gender	Male	12 (6.25)
	Female	180 (93.75)
Education	Illiterate	66 (34.38)
	Primary school	72 (37.50)
	Secondary/high school	34 (17.71)
	Higher secondary and above	20 (10.42)
Occupation	Unskilled	42 (21.88)
	Service	18 (9.38)
	Housewives	132 (68.75)
Socioeconomic status*	Upper	30 (15.63)
	Upper middle	18 (9.38)
	Lower middle	24 (12.50)
	Upper lower	30 (15.63)
	Lower	90 (46.88)
Religion	Hindu	162 (84.38)
	Muslim	12 (6.25)
	Christian	18 (9.38)
Residence	Urban	48 (25.00)
	Rural	144 (75.00)
Food habits	Vegetarian	36 (18.75)
	Non-vegetarian	156 (81.25)
Addiction**	Betel-nut chewing	138 (71.90)
	Smoking	12 (6.30)
	Alcohol consumption	12 (6.30)
	No addiction	42 (21.90)
Previous history of cholecystitis	Yes	78 (40.60)
	No	114 (59.40)
Diabetes mellitus	Yes	24 (12.50)
	No	168 (87.50)
Hypertension	Yes	48 (25.00)
	No	144 (75.00)
Family history of cancer	Yes	24 (12.50)
	No	168 (87.5)

**Figure 1:**
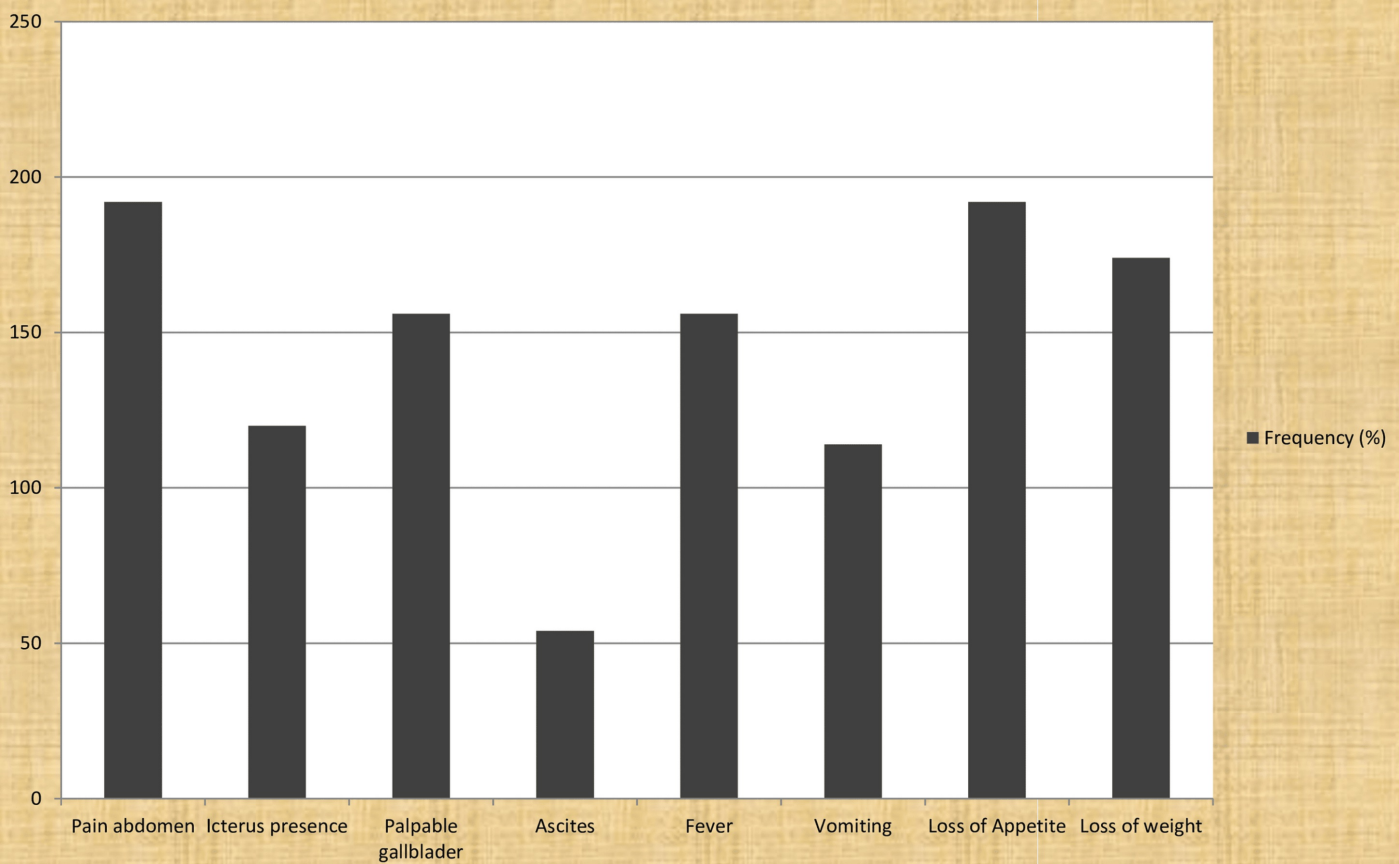
Clinical profile of patients with GBC in Tripura (n = 192) showing abdominal pain and loss of appetite as the most common presenting symptoms GBC: gallbladder carcinoma

Clinical presentation was dominated by abdominal pain and loss of appetite, both of which were reported by all patients. Significant weight loss was present in 90.6% of cases. On clinical examination, fever and a palpable gallbladder were observed in 81.3% of patients, whereas jaundice and ascites were noted in 62.5% and 28.1% of patients, respectively (Table 2, Figure 1).

**Table 2:** Clinical profile of patients with GBC in Tripura (n = 192) * Patients may have presented with more than one clinical feature; therefore, percentages do not sum to 100%. GBC: gallbladder carcinoma

Clinical findings^*^		Frequency (%)
Symptoms	Abdominal pain	192 (100)
	Loss of appetite	192 (100)
	Weight loss	174 (90.6)
	Vomiting	114 (59.4)
Clinical signs	Jaundice	120 (62.5)
	Palpable gallbladder	156 (81.3)
	Fever	156 (81.3)
	Ascites	54 (28.1)

**Figure 2:**
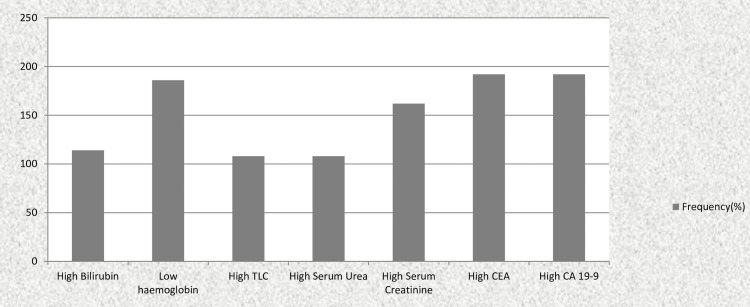
Laboratory investigation findings of patients with GBC in Tripura (n = 192) The figure shows that 96.9% of patients had low hemoglobin levels, 59.4% had elevated serum bilirubin, and 100% had elevated tumor markers, including CEA and CA 19-9. CEA: carcinoembryonic antigen, CA 19-9: carbohydrate antigen 19-9, GBC: gallbladder carcinoma

Dietary and lifestyle assessment revealed that 156 patients (81.2%) followed a non-vegetarian diet, and 138 (71.9%) reported betel-nut chewing as a habitual addiction (Table 1). Laboratory findings among the participants showed that 186 (96.9%) had low hemoglobin, 114 (59.4%) had high bilirubin, and 192 (100%) had high CEA and CA 19-9 (Table 3, Figure 2).

**Table 3:** Laboratory investigation findings of patients with GBC in Tripura (n = 192) ^+ ^Values were classified according to the laboratory reference range used at the study institutions. ^# ^Reference ranges are shown in the table. Percentages may not total 100% because of rounding. CEA: carcinoembryonic antigen, CA 19-9: carbohydrate antigen 19-9, GBC: gallbladder carcinoma

Laboratory parameter#	Reference range^+^	Frequency (%)
Serum bilirubin (mg/dL)	Normal (<1.2)	78 (40.6)
	Elevated (>1.2)	114 (59.4)
Hemoglobin (g/dL)	Within reference range	6 (3.1)
	Below reference range	186 (96.9)
Total leukocyte count (cells/µL)	Normal (<11,000)	84 (43.8)
	Elevated (>11,000)	108 (56.3)
Serum urea (mg/dL)	Normal (<20)	108 (56.3)
	Elevated (>20)	84 (43.8)
Serum creatinine (mg/dL)	Normal (<1.5)	162 (84.4)
	Elevated (>1.5)	30 (15.6)
CEA (ng/mL)	Normal (0-2.5)	0 (0)
	Elevated (>2.5)	192 (100)
CA 19-9 (U/mL)	Normal (0-39)	0 (0)
	Elevated (>39)	192 (100)

Radiological assessment demonstrated a gallbladder mass on ultrasonography in 56.3% of patients, while 34.3% had a gallbladder mass associated with calculi. Periportal lymphadenopathy was identified on ultrasonography in 9.4% of patients. On CECT, local hepatic invasion was the most common imaging finding (43.8%), followed by hepatic metastases (15.6%) and associated gallstones (15.6%). CECT reports were unavailable in 25.0% of cases (Table 4).

**Table 4:** Radiological findings, cytopathological diagnosis, and clinical stage at presentation of patients with GBC in Tripura (n = 192) $ Radiological findings were recorded according to the predominant imaging feature documented in patient records. * Clinical staging was assigned using available radiological findings and AJCC 8th edition criteria. Pathological staging was unavailable in many patients because definitive surgical resection was not performed. GBC: gallbladder carcinoma, AJCC: American Joint Committee on Cancer

Investigation	Findings^$^	Frequency (%)
USG whole abdomen	Mass arising from gallbladder	108 (56.3)
	Gallbladder mass with calculi	66 (34.3)
	Peri-portal lymph nodes	18 (9.4)
CECT whole abdomen	Malignant gallbladder mass with local hepatic invasion	84 (43.8)
	Gallbladder mass with hepatic secondaries	30 (15.6)
	Gallbladder mass with calculi	30 (15.6)
	Report not available	48 (25.0)
Cytopathological diagnosis among included patients	Well-differentiated adenocarcinoma	78 (40.63)
	Moderately differentiated adenocarcinoma	42 (21.88)
	Poorly differentiated adenocarcinoma	48 (25.00)
	Undifferentiated/anaplastic carcinoma	24 (12.50)
Clinical stage at presentation (AJCC 8th edition)*	Stage IIA	48 (25.0)
	Stage IIIA	24 (12.5)
	Stage IIIB	30 (15.6)
	Stage IVA	72 (37.5)
	Stage IVB	18 (9.4)

As cytological confirmation was an inclusion criterion, all patients underwent image-guided FNAC. Adenocarcinoma represented the predominant cytological subtype, accounting for 87.5% of cases. Among these, well-differentiated adenocarcinoma was the most common subtype (40.6%), followed by poorly differentiated (25.0%) and moderately differentiated adenocarcinoma (21.9%). Undifferentiated/anaplastic carcinoma was identified in 12.5% of patients.

Clinical staging based on the AJCC 8th edition criteria revealed that no patients met the criteria for stage I, IB, or IIB disease at presentation. Most patients presented with advanced disease. Stage IVA was the most common stage at diagnosis (37.5%), followed by stage IIA (25.0%), stage IIIB (15.6%), stage IIIA (12.5%), and stage IVB (9.4%). Overall, 75.0% of patients presented with stage III or stage IV disease (Table 4, Figure 1).

**Figure 3:**
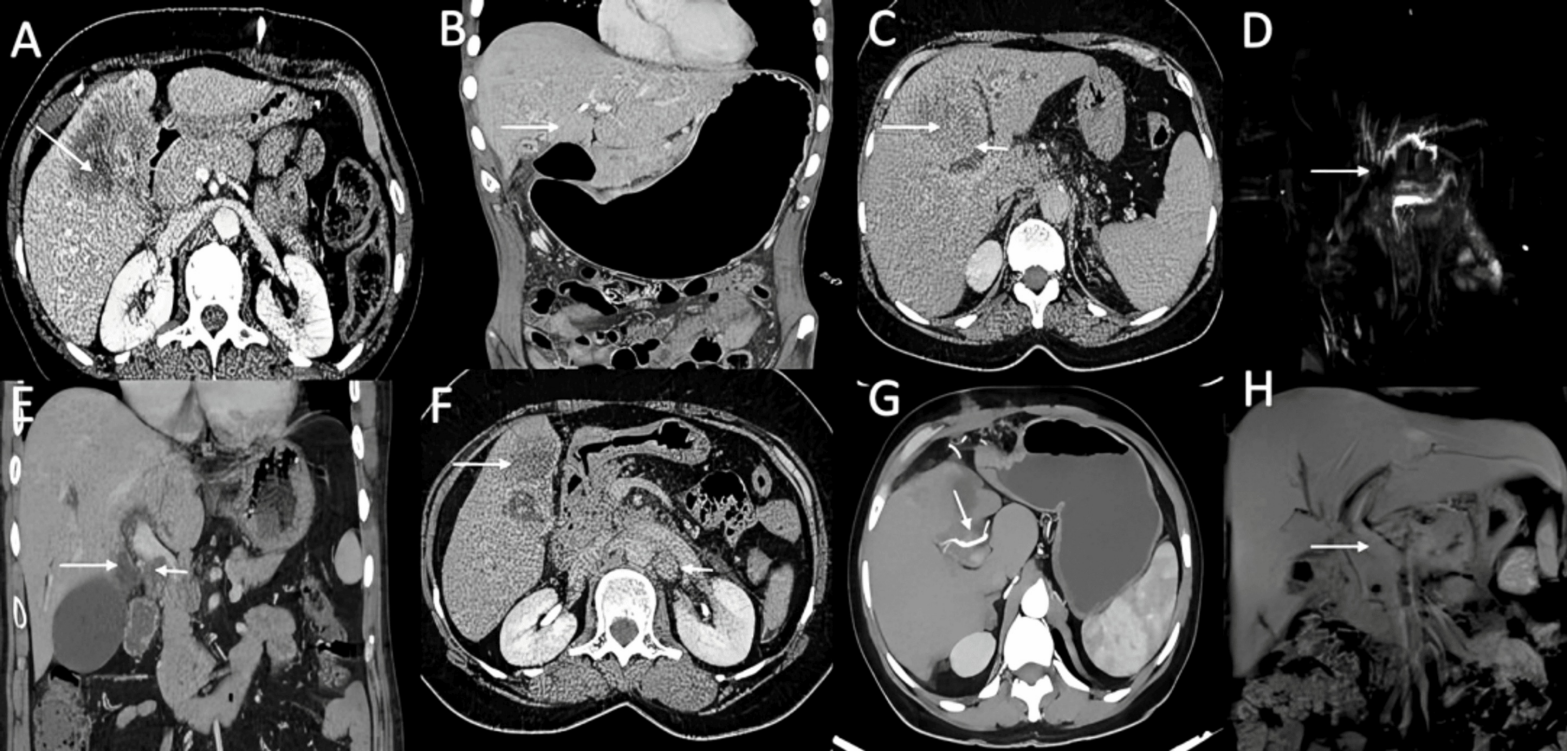
CECT findings in GBC Axial and coronal CECT images (A-H) demonstrate a heterogeneously enhancing gallbladder mass (arrows) with direct invasion of adjacent hepatic segments IVb and V, loss of the fat plane between the gallbladder and liver, and local hepatic infiltration. Associated findings include gallbladder wall thickening, regional lymphadenopathy, and features suggestive of advanced-stage disease. These radiological findings are consistent with locally advanced GBC. GBC: gallbladder carcinoma, CECT: contrast-enhanced computed tomography

## Discussion

In the present study, we evaluated the demographic, clinical, radiological, and cytopathological profile of 192 patients with GBC managed at tertiary-care referral centers in Tripura, a northeastern state of India. Although GBC is relatively uncommon worldwide, its incidence demonstrates substantial geographic, ethnic, and socioeconomic variation, with particularly high disease burden reported from parts of South America and the northern and northeastern regions of India [4]. The present study contributes region-specific data from Tripura, where published information on the clinicodemographic characteristics of GBC remains limited.

The incidence of GBC increases with advancing age. In our cohort, the mean age at presentation was 61.3 ± 6.1 years, and more than half of the patients were older than 60 years. These findings are broadly consistent with previous Indian studies reporting a peak incidence in the sixth and seventh decades of life [5]. A marked female predominance was observed, with women accounting for 93.8% of cases. This finding is consistent with both Indian and international literature demonstrating a substantially higher incidence among females, particularly in North India, Pakistan, and American Indian populations [6]. Hormonal influences, gallstone disease, reproductive factors, and metabolic risk factors have been proposed as potential contributors to this gender disparity. The remarkably high female predominance observed in our study may also reflect regional demographic characteristics, referral patterns, or healthcare-seeking behavior and warrants further investigation in larger population-based studies.

Socioeconomic and geographic factors may influence healthcare access and stage at diagnosis. In our study, three-fourths of patients belonged to lower-middle or lower socioeconomic strata, comparable to observations reported by Dubey et al. [3]. Similarly, 75.0% of patients resided in rural areas. This contrasts with the findings of Saikia et al., who reported a predominance of urban cases in Dibrugarh [9]. The high proportion of rural patients in our cohort may reflect regional population distribution and contribute to delayed access to healthcare, reduced availability of specialist services, and late-stage presentation. However, the descriptive design of the present study does not permit inference regarding socioeconomic status or rural residence as independent risk factors for disease occurrence.

Dietary and lifestyle characteristics observed in the present study merit attention. A large majority of patients reported a non-vegetarian diet. Previous epidemiological studies have suggested associations between dietary practices, including the consumption of animal fat and certain cooking oils, and the risk of GBC [10]. Additionally, 12.5% of patients had diabetes mellitus, a condition previously associated with gallstone disease and an increased risk of GBC [11]. Nevertheless, because the present study lacked a comparison group, these observations should be interpreted as descriptive findings rather than evidence of causal associations.

The clinical presentation of GBC in our cohort largely reflected advanced disease at the time of diagnosis. Abdominal pain was the most common presenting symptom and was reported by all patients, consistent with previous studies reporting pain in 81-100% of cases [5,6]. Jaundice was present in 62.5% of patients, exceeding the 35.8% prevalence reported by Gupta et al. [12]. Similarly, a palpable gallbladder was identified in 81.3% of patients, substantially higher than rates reported in several previous series. Comparable findings were reported by Dubey et al., who observed abdominal pain in all patients and obstructive jaundice in 51.4% of cases [3]. The high prevalence of jaundice and palpable gallbladder in our cohort likely reflects the predominance of locally advanced disease at presentation. These findings underscore the diagnostic challenge posed by GBC, as early symptoms are often nonspecific and may mimic benign hepatobiliary disorders.

Radiological imaging remains the cornerstone of the initial evaluation of suspected GBC, while image-guided FNAC provides pathological confirmation in unresectable or advanced disease [13]. In our study, ultrasonography identified a gallbladder mass in 56.3% of patients, comparable to the 51% detection rate reported by Gupta et al. [12]. CECT frequently demonstrated local hepatic invasion and metastatic disease, highlighting the advanced stage at which many patients presented. Cytopathological evaluation revealed adenocarcinoma in 87.5% of cases, consistent with the established histological spectrum of GBC reported in previous studies [13]. Well-differentiated adenocarcinoma accounted for 40.6% of tumors, a proportion higher than that reported by Dubey et al. [3], which may reflect differences in referral patterns, disease biology, or sampling characteristics.

Laboratory abnormalities were common in our study population and included anemia, hyperbilirubinemia, leukocytosis, and elevated tumor-marker levels. The uniformly elevated levels of CEA and CA 19-9 should be interpreted cautiously. As the present study included only cytologically confirmed cases managed at tertiary-care referral centers, the observed prevalence of elevated tumor markers may reflect referral bias toward patients with clinically significant or advanced disease. It should not be generalized to all patients with GBC [14].

An important finding of this study was the predominance of advanced-stage disease at presentation. Clinical staging based on AJCC eighth edition criteria showed that 75% of patients presented with stage III or stage IV disease. In contrast, only one-fourth had stage II disease, and no patients met criteria for stage I disease. These findings are consistent with those reported by Batra et al., who observed early-stage disease in only a small proportion of patients [15]. The predominance of advanced-stage presentation likely reflects a combination of nonspecific early symptoms, low disease awareness, delayed healthcare-seeking behavior, and limited opportunities for early detection. Because curative treatment is largely restricted to early-stage disease, delayed presentation continues to represent a major challenge in improving outcomes for patients with GBC [6,14].

The findings of this study should be interpreted in light of certain limitations. The study was conducted at tertiary-care referral centers and is therefore susceptible to referral bias. Pathological staging was unavailable in many patients because definitive surgical resection was not performed, necessitating reliance on radiology-based clinical staging. Furthermore, the cross-sectional design precludes assessment of temporal relationships or causal associations between demographic and lifestyle factors and disease occurrence. Despite these limitations, the study provides valuable contemporary data on the demographic, clinical, radiological, and cytopathological characteristics of patients with GBC from an underrepresented, high-burden region of North-East India.

## Conclusions

This study provides contemporary evidence on the demographic, clinical, radiological, and cytopathological characteristics of patients with GBC in Tripura, a high-burden region of North-East India. GBC predominantly affected elderly women and was more commonly observed among individuals from rural and lower socioeconomic backgrounds. Adenocarcinoma was the predominant cytopathological subtype, and three-fourths of patients presented with stage III or stage IV disease.

The predominance of advanced-stage presentation highlights the persistent challenge of delayed diagnosis in GBC, largely due to nonspecific clinical manifestations and barriers to timely healthcare access. Strengthening public awareness, improving diagnostic infrastructure, and facilitating early referral to specialized centers may help promote earlier detection and potentially improve outcomes. Further multicentric studies from North-East India are warranted to better characterize regional disease patterns and to guide targeted prevention and early detection strategies.
